# No replication of previously reported association with genetic variants in the T cell receptor alpha (TRA) locus for myalgic encephalomyelitis/chronic fatigue syndrome (ME/CFS)

**DOI:** 10.1038/s41398-022-02046-1

**Published:** 2022-07-11

**Authors:** Marthe Ueland, Riad Hajdarevic, Olav Mella, Elin B. Strand, Daisy D. Sosa, Ola D. Saugstad, Øystein Fluge, Benedicte A. Lie, Marte K. Viken

**Affiliations:** 1 Department of Medical Genetics, Oslo University Hospital and University of Oslo, Oslo, Norway; 2 Institute of Clinical Medicine, University of Oslo, Oslo, Norway; 3 Department of Oncology and Medical Physics, Haukeland University Hospital, Bergen, Norway; 4 Department of Clinical Science, University of Bergen, Bergen, Norway; 5 Faculty of Health, VID Specialized University, Oslo, Norway; 6 Department of Digital Health Research, Division of Medicine, Oslo University Hospital, Oslo, Norway; 7 CFS/ME Center, Oslo University Hospital, Oslo, Norway; 8 National Advisory Unit on CFS/ME, Oslo, Norway; 9 Department of Pediatric Research, Oslo University Hospital, University of Oslo, Oslo, Norway; 10 Department of Immunology, Rikshospitalet, Oslo University Hospital and University of Oslo, Oslo, Norway

**Keywords:** Genetics, Diseases

## Abstract

Myalgic encephalomyelitis/chronic fatigue syndrome (ME/CFS) is a disease with a variety of symptoms such as post-exertional malaise, fatigue, and pain, but where aetiology and pathogenesis are unknown. An increasing number of studies have implicated the involvement of the immune system in ME/CFS. Furthermore, a hereditary component is suggested by the reported increased risk for disease in relatives, and genetic association studies are being performed to identify potential risk variants. We recently reported an association with the immunologically important human leucocyte antigen (HLA) genes *HLA-C* and *HLA-DQB1* in ME/CFS. Furthermore, a genome-wide genetic association study in 42 ME/CFS patients reported significant association signals with two variants in the T cell receptor alpha (TRA) locus (*P* value <5 × 10^−8^). As the T cell receptors interact with the HLA molecules, we aimed to replicate the previously reported findings in the TRA locus using a large Norwegian ME/CFS cohort (409 cases and 810 controls) and data from the UK biobank (2105 cases and 4786 controls). We investigated numerous SNPs in the TRA locus, including the two previously ME/CFS-associated variants, rs11157573 and rs17255510. No associations were observed in the Norwegian cohort, and there was no significant association with the two previously reported SNPs in any of the cohorts. However, other SNPs showed signs of association (*P* value <0.05) in the UK Biobank cohort and meta-analyses of Norwegian and UK biobank cohorts, but none survived correction for multiple testing. Hence, our research did not identify any reliable associations with variants in the TRA locus.

## Introduction

Myalgic encephalomyelitis/chronic fatigue syndrome (ME/CFS) is a disabling disease estimated to affect from 0.2 to 2% of the population, depending on the diagnostic criteria used [1]. No biomarkers exist [2]. Thus, diagnosis is based on the presence of core symptoms (e.g. post-exertional malaise, fatigue and pain) and exclusion of other possible illnesses using one of the proposed diagnostic criteria, such as Canadian Consensus Criteria, International Consensus Criteria or Fukuda Criteria [3–5]. The aetiology and pathogenesis of ME/CFS are poorly understood, however, there is increasing evidence pointing towards an immunological component. Elevated levels of autoantibodies against ß2 adrenergic receptors, and muscarinic 3 and 4 acetylcholine receptors have been reported [6, 7]. Additionally, decreased cytotoxicity of CD8+ T cells [8] and increased presence of autoreactive T cells [9] have also been observed in ME/CFS patients.

A heritable component is implicated by the reported increased risk in relatives of ME/CFS patients [10–12], and genetic association studies are emerging in order to identify risk variants. We have recently reported associations with specific HLA alleles, HLA-C*07:04 and HLA-DQB1*03:03 [13], and individuals carrying either one or both risk alleles seem to more often respond positively to the immunosuppressive drug cyclophosphamide [14]. The HLA locus is well-documented as the main genetic determinant in other immune-mediated diseases, such as multiple sclerosis and rheumatoid arthritis [15]. The functional role of the HLA molecules in presenting peptides to the T cell receptors (TCRs), also makes the latter genes prime candidates for involvement in disease predisposition.

The TCR diversity is driven by somatic recombination between different variable (V), diversity (D) and joining (J) gene segments, a process guided by recombination signal sequences (RSS) [16]. SNPs located in the RSS have been suggested to influence the gene usage during the V(D)J recombination [17], and thus also affect the TCR repertoire.

Genetic variation within the TCR locus encoding the T cell receptor alpha (TRA) has previously been found to be associated with multiple sclerosis [18] and narcolepsy [19]. Furthermore, Schlauch et al reported an association between two SNPs (rs11157573 and rs17255510) in the TRA locus and ME/CFS in a study of 42 patients and 38 controls [20]. Given these previously reported TRA associations and the functional relevance of the TCR, we have investigated numerous SNPs across the TRA locus in large ME/CFS cohorts.

## Materials and methods

### Study population

In this study, 409 Norwegian ME/CFS patients diagnosed according to the Canadian Consensus Criteria [4] and 810 healthy, ethnically matched controls obtained from the Norwegian Bone Marrow Donor Register were included. The gender distribution was 336 females (82%) and 73 males (18%) in ME/CFS patients, and 448 females (55%) and 362 males (45%) in controls. All patients have previously been included in association studies of the HLA region and have given informed consent [13, 21], however, only patients with DNA currently available were included in this study. The research project was approved by the Regional Committees for Medical and Health Research Ethics (REK, 2015/1547). Genotyping data for the TRA region extracted from 2105 individuals registered with the field code 1182 (chronic fatigue syndrome) and 4786 gender and ethnically matched, randomly selected controls from the UK biobank were also included. The selection of UK biobank cases and controls was performed as part of another study and included a principal component analysis to match cases and controls [22]. Access to the UK biobank data was granted through UK biobank application 43949, and we complied with all relevant ethical regulations for the UK biobank.

### Genotyping

Genotyping data for 27 SNPs in the TRA locus (chr14:21,870,000-23,500,000; GRChr37) were extracted for the cases and controls from previously genotyped array datasets [21, 23, 24], generated using Infinium ImmunoArray-24 v2 BeadChip and HumanImmuno-v1 BeadChip (Illumina, San Diego, USA). In addition, three SNPs, not present in the array dataset, were genotyped using Taqman SNP Genotyping Assays (Thermo Fisher Scientific, Waltham, MA, USA). These were, rs11157573 (C__32009806_10) and rs17255510 (C__34374423_10) previously reported to be associated with ME/CFS [20], and rs35379740 (C__25962246_10) located in the recombination signal sequence of TRAV14DV4. Genotyping was conducted according to the manufacturer using TaqPath™ ProAmp™ Master Mix (Thermo Fisher Scientific) with 5 ng genomic DNA as input and analysed on a QuantStudio™ 12 K Flex instrument (Thermo Fisher Scientific).

HLA carrier status for HLA-C*07:04 and HLA-DQB1*03:03 was available for all ME/CFS patients [13, 21].

### Quality control

For the Taqman genotyping (AppliedBiosystems), allelic discrimination plots were inspected manually to ensure well-separated clusters and good quality allele-calls. We only allowed SNPs with a genotype success rate >95% and being in Hardy–Weinberg equilibrium (*P* value >0.001) to be included in our association analyses.

### Power calculations

Our study had more than 80% power to detect genotype risk ratios >1.33 for a multiplicative or additive disease model, respectively, using the Genetic Association Study (GAS) power calculator (https://csg.sph.umich.edu/abecasis/gas_power_calculator/) with the settings: significance level = 0.05, prevalence = 0.02 and allele frequencies from 0.27 in the Norwegian cohort.

### Statistical methods

Plink v1.9 [25] was used for Hardy–Weinberg equilibrium test (HWE), and for association analysis with one degree of freedom chi-square allelic test both between ME/CFS patients and controls and between HLA-risk and HLA-non-risk patients. For the meta-analysis, Plink performs basic fixed effects and random effects. Linkage disequilibrium (LD) plots were generated by Haploview v4.2 [26]. LocusZoom [27] was used to make combined association and LD plots. Unphased v3.0.13 [28] was used for two SNP haplotype analysis for neighbouring markers using a rare frequency threshold of 1% in either cases or controls. Correction for multiple testing was performed using Bonferroni.

## Results

The genotyping success rate was >99.7% across all 30 SNPs, and no significant deviation from Hardy–Weinberg equilibrium was observed. ME/CFS patients and controls displayed a similar linkage disequilibrium (LD) pattern across the TRA locus, with few SNPs showing strong LD (r^2^ > 0.90, supplementary Fig. 1).

**Fig. 1:**
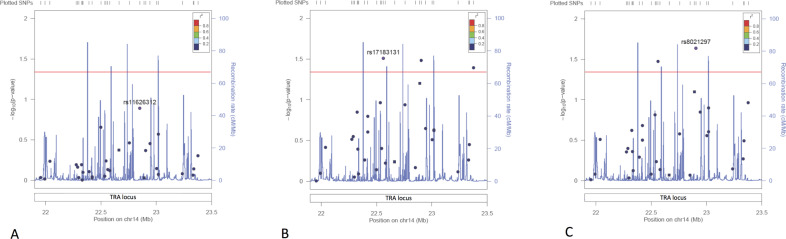
Association analyses of the TRA locus. LocusZoom plot of the 30 SNPs covering the TRA locus from **A** the Norwegian cohort, **B** the UK Biobank cohort and **C** meta-analysis of the Norwegian and UK biobank cohorts. The y axis is showing the –log10(*P* value) while the x-axis is showing the base pair position on chromosome 14. SNPs are marked as dots and recombination rate by blue lines on the plot. Notably, the previously associated SNPs, rs17255510 and rs11157573, are marked as squares. The colour of the SNPs indicates the LD measured in *r*^2^. The red line represents *P* value = 0.05.

We first analysed the Norwegian ME/CFS cohort, where the patients had been diagnosed according to the Canadian Consensus Criteria (409 cases and 810 controls). Association analyses of the 30 SNPs across the TRA locus did not show any significant differences (*P* value >0.05) in allele frequencies between the Norwegian ME/CFS patient and controls (Fig. 1A and supplementary Table 1). Nor were there any significant differences between males and females. As the TRA locus is a complex genetic region, single SNP associations might not detect potential association signals. Thus, we next performed two-locus haplotype analyses, which did not show signs of associations (*P* value >0.05, the top hit was for rs2001022 and rs11626312 with a *P* value = 0.07).

Given the biological interactions between HLA and TCR, one could hypothesise that the TRA association could be more evident in the subset of ME/CFS patients carrying the HLA-risk alleles (HLA-C*07:04 and/or -DQB1*03:03). However, no significant differences were observed for the TRA allele frequencies between Norwegian ME/CFS patients being HLA-risk allele carriers vs non-carriers.

Next, we also investigated the same 30 SNPs using a cohort of self-reported CFS patients (2105 cases and 4786 controls) from the UK biobank. The two previously ME/CFS-associated SNPs, rs11157573 and rs17255510 showed no signs of association in the UK cohort either (Fig. 1B). However, three SNPs had a *P* value <0.05 (rs17183131, rs8021297 and rs8005677, Fig. 1B). In a meta-analysis of both the Norwegian and UK cohort, two of these SNPs, rs8021297 (*P* value = 0.02) and rs17183131 (*P* value = 0.03), still showed weak signs of association (Fig. 1C). Stratifying for HLA-risk allele carriers versus non-carriers in the combined Norwegian (73 carriers, 336 non-carriers) and UK dataset (294 carriers, 1811 non-carriers) displayed only one SNP, rs8572 (OR = 0.76, *P* value = 0.03), with *P* value <0.05 (Supplementary Table 1). None of these SNPs reached a Bonferroni corrected significance threshold of *P* value <0.002 when correcting for the 30 SNPs tested.

**Fig. 2:**
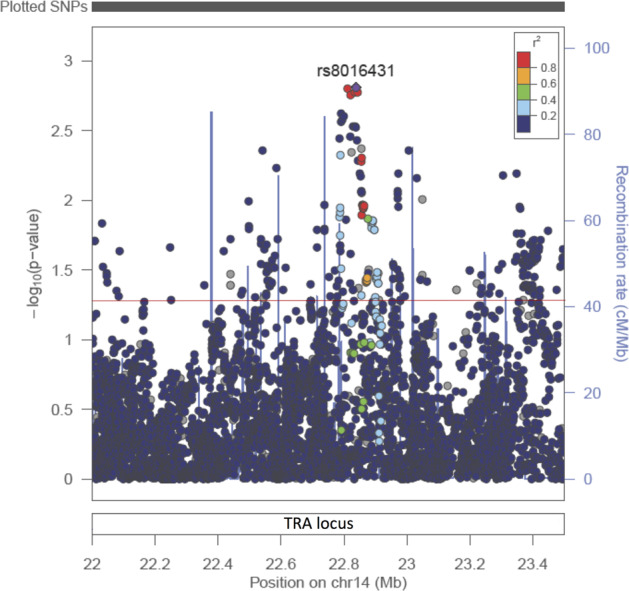
LocusZoom plot of all SNPs (*N* = 6769, including imputed SNPs) in the TRA locus for 2105 ME/CFS cases and 4786 controls from the UK biobank. The y axis is showing the –log10(*P* value) while the x-axis is showing the base pair position on chromosome 14. SNPs are marked as dots and recombination rate by blue lines on the plot. The colour of the SNPs is indicating LD measured in *r*^2^. The red line represents *P* value = 0.05.

When including all SNPs (*N* = 6769) in the TRA region from the UK Biobank dataset, there were 272 SNPs showing signs of association (*P* value <0.05), with the most significant being rs8016431 (OR = 0.88, 95% CI (0.80–0.95), *P* value = 0.0015, Fig. 2). However, these had a *P* value that could not withstand correction for multiple testing (*P* value <7.4 × 10^−6^).

The SNPs, rs11157573 and rs17255510, previously reported by Schlauch et al. to be ME/CFS-associated [20], had similar allele frequencies in the Norwegian and the UK biobank datasets (Table 1). These frequencies did not deviate between cases and controls (*P* value >0.05). Furthermore, these frequencies were similar to that seen in the 1000 Genomes CEU dataset. The control frequencies reported by Schlauch et al were comparable to ours, albeit somewhat lower. However, the large deviation between the datasets was their highly increased case frequencies (Table 1), which is driving their reported association.

**Table 1:** Overview of the allele frequencies for rs17255510 and rs11157573 observed in our Norwegian 409 ME/CFS cases and 810 controls, the UK biobank with 2105 ME/CFS cases and 4786 controls compared to frequencies from the previous publication by ref. [20], and the 1000 genomes project (CEU; Northern Europeans from Utah) [33].

		Our study		UK biobank		Schlauch et al.		1000 G CEU
SNPs	Allele	Cases	Controls	Cases	Controls	Cases	Controls	General
rs17255510	C	0.220	0.218	0.214	0.234	0.679	0.171	0.232
rs11157573	C	0.180	0.184	0.171	0.178	0.488	0.158	0.197

## Discussion

We have in the current study screened the TRA locus for genetic association with ME/CFS in a large dataset, without identifying any significant association. More specifically, we did not replicate the previously reported association between rs17255510 and rs11157573 and ME/CFS [20]. Our stratified analysis of patients carrying HLA-risk alleles and non-carriers did not indicate any significant associations with TRA.

The control frequencies reported by Schlauch et al. were comparable to that observed in our control datasets from Norway and the UK, as well as in the CEU cohort from the 1000 Genomes. However, Schlauch et al reported vastly increased case frequencies compared to what we observed for rs17255510 and rs11157573 (see Table 1). Differences in allele frequencies could be due to different ethnicities, however, the ethnicity of the cohort investigated by Schlauch et al. is not stated in their article. Furthermore, the magnitude of the differences in frequencies they observed between their cases and controls is far beyond what is generally reported in complex diseases.

Other factors might have influenced the discrepancies between our studies. Clinical manifestations in ME/CFS cases are known to be heterogeneous [29]. Both Schlauch et al. (*N* = 42) and our Norwegian (*N* = 409) ME/CFS patients met the Canadian Consensus Criteria for diagnosis, while the patients (*N* = 2105) from the UK cohort were self-reported. Furthermore, the variation in sample size between the cohorts affect their statistical power. As an example, our study only had 80% power to detect associations at a significance level of 0.05 for OR >1.33, while a study with 100 cases and 100 controls (twice the size of the Schlauch et al. study) using the same calculation settings only have ~27% statistical power. Studies with few individuals are more affected by random effects like selection bias and genotyping errors [30]. Hence, increasing the chance of false-positive discoveries.

The sample size of our study is larger than that of Schlauch et al., but nevertheless suboptimal to detect significance for low effect sizes typically seen for complex diseases [31]. Studies unable to replicate an initially reported association often show consistent results with the initial observation, but lack the power to detect it [32]. A 1% difference in allele frequencies between cases and controls was seen for some of the tested SNPs in our dataset, which, if representing a genuine difference, could reach significance in a much larger population. Hence, the lack of association in this study cannot be used to conclude that TRA is not involved in the pathogenesis of ME/CFS. This is underpinned by the signs of association observed in the self-reported UK cohort, which did not withstand correction for multiple testing. Further studies and meta-analyses are necessary to either confirm or reject an association between TRA and ME/CFS. Reliable findings will be achieved both by including more patients and controls as well as a denser set of SNPs across the TRA region. The DecodeME initiative (https://www.decodeme.org.uk/), with the goal of recruiting 20,000 ME/CFS patients, will hopefully enable robust association studies with good statistical power.

Taken together, the current study, which to date represents by far the largest study addressing the association between the TRA locus and ME/CFS, could not find any association.

## Supplementary information

Supplementary figure 1

Supplementary table 1
