# Supplementary material for: No replication of previously reported association with genetic variants in the T cell receptor alpha (TRA) locus for myalgic encephalomyelitis/chronic fatigue syndrome (ME/CFS)
Source: Transl Psychiatry. 2022 Jul 11;12:277. doi: 10.1038/s41398-022-02046-1 (PMC9276688; doi:10.1038/s41398-022-02046-1)

Supplementary figure 1. Linkage disequilibrium (LD) plots of the 30 SNPs in TRA on chromosome 14 in 409 Norwegian ME/CFS cases and 810 Norwegian controls. The darkness of color corresponds to the measure of LD (D' or r^2), with the darkest correlating to the highest LD. LD measured as **A)** D' in ME/CFS cases, **B)** D' in controls, **C)** r^2 in ME/CFS cases and **D)** r^2 in controls.

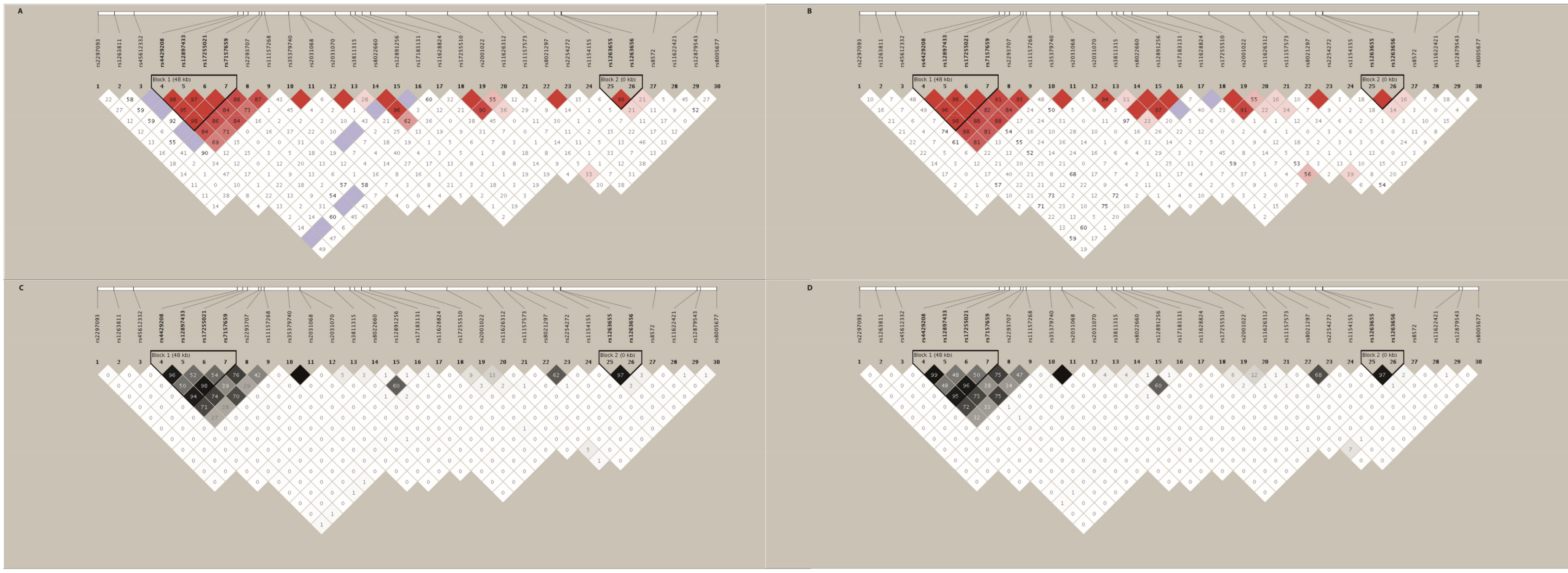

Supplement: Supplementary file 1 — Supplementary figure 1 [file 41398_2022_2046_MOESM1_ESM.pdf]
