# Supplementary material for: No replication of previously reported association with genetic variants in the T cell receptor alpha (TRA) locus for myalgic encephalomyelitis/chronic fatigue syndrome (ME/CFS)
Source: Transl Psychiatry. 2022 Jul 11;12:277. doi: 10.1038/s41398-022-02046-1 (PMC9276688; doi:10.1038/s41398-022-02046-1)
Supplement: Supplementary file 2 — Supplementary table 1 [file 41398_2022_2046_MOESM2_ESM.docx]

Supplementary table 1: Association analyses of 409 Norwegian ME/CFS cases and 810 controls, and an HLA-risk stratified analysis of 73 ME/CFS patients having one or both of the potential HLA risk alleles, HLA-C*07:04 and HLA-DQB1*03:03, versus 336 ME/CFS cases having neither allele. A meta-analysis of HLA-risk stratified analyses of the same 409 Norwegian ME/CFS patients and 2105 ME/CFS patients from the UK biobank, where 294 UK ME/CFS patients have one or both HLA-risk alleles and 1811 ME/CFS cases have neither allele. Positions are according to build 37, MAF is minor allele frequency, OR is odds ratio, 95% CI is the 95% confidence interval. SNPs reported to be associated by Schlauch et al [20], are marked in bold.

|  |  | **Association analysis of 409 ME/CFS cases and 810 controls** | | | | **Stratified analysis for HLA-C*07:04 and/or -DQB1*03:03 carriers versus non-carriers of the ME/CFS patients** | | **Meta-analysis of HLA-C*07:04 and/or -DQB1*03:03 carriers versus non-carriers in Norwegian and UK ME/CFS patients** | |
| --- | --- | --- | --- | --- | --- | --- | --- | --- | --- |
| **SNP** | **Position** | **MAF cases** | **MAF controls** | **P-value** | **OR (95 % CI)** | **P-value** | **OR (95 % CI)** | **P-value** | **OR** |
| rs2297093 | 21955356 | 0.449 | 0.451 | 0.93 | 0.99 (0.84-1.18) | 0.93 | 0.98 (0.69-1.41) | 0.71 | 0.97 |
| rs1263811 | 21993498 | 0.116 | 0.115 | 0.96 | 1.01 (0.77-1.31) | 0.58 | 0.85 (0.47-1.52) | 0.49 | 1.08 |
| rs45612332 | 22038562 | 0.042 | 0.037 | 0.58 | 1.13 (0.73-1.73) | 0.16 | 0.43 (0.13-1.44) | 0.45 | 1.14 |
| rs4429208 | 22276339 | 0.069 | 0.064 | 0.65 | 1.08 (0.77-1.52) | 1.00 | 1.00 (0.49-2.03) | 0.34 | 1.16 |
| rs12897433 | 22289606 | 0.069 | 0.064 | 0.69 | 1.07 (0.77-1.50) | 0.72 | 0.87 (0.42-1.83) | 0.31 | 1.16 |
| rs17255021 | 22299981 | 0.117 | 0.116 | 0.92 | 1.01 (0.78-1.32) | 0.37 | 0.76 (0.42-1.39) | 0.79 | 1.03 |
| rs7157659 | 22325140 | 0.067 | 0.062 | 0.64 | 1.08 (0.77-1.52) | 0.77 | 0.89 (0.43-1.87) | 0.27 | 1.18 |
| rs2293707 | 22331057 | 0.069 | 0.069 | 1.00 | 1.00 (0.72-1.39) | 0.28 | 0.64 (0.28-1.44) | 0.16 | 1.22 |
| rs11157268 | 22338141 | 0.116 | 0.120 | 0.79 | 0.97 (0.74-1.25) | 0.09 | 0.57 (0.30-1.10) | 0.79 | 1.03 |
| rs35379740 | 22392838 | 0.053 | 0.051 | 0.78 | 1.06 (0.72-1.54) | 0.75 | 0.87 (0.38-2.00) | 0.85 | 1.03 |
| rs2031068 | 22420778 | 0.201 | 0.202 | 0.94 | 0.99 (0.80-1.22) | 0.77 | 0.94 (0.60-1.47) | 0.40 | 1.09 |
| rs2031070 | 22420885 | 0.199 | 0.201 | 0.90 | 0.99 (0.80-1.22) | 0.81 | 0.95 (0.60-1.49) | 0.42 | 1.08 |
| rs3811315 | 22499870 | 0.115 | 0.099 | 0.22 | 1.18 (0.90-1.55) | 0.82 | 0.94 (0.53-1.66) | 0.74 | 1.05 |
| rs8022660 | 22535262 | 0.296 | 0.299 | 0.89 | 0.99 (0.82-1.19) | 0.66 | 0.92 (0.62-1.36) | 0.72 | 0.97 |
| rs12891256 | 22545787 | 0.480 | 0.469 | 0.58 | 1.05 (0.89-1.24) | 0.57 | 0.90 (0.63-1.29) | 0.40 | 0.94 |
| rs17183131 | 22562099 | 0.020 | 0.022 | 0.74 | 0.90 (0.50-1.64) | 0.92 | 1.06 (0.30-3.78) | 0.48 | 1.20 |
| rs11628824 | 22580762 | 0.413 | 0.420 | 0.76 | 0.97 (0.82-1.15) | 0.76 | 1.06 (0.74-1.52) | 0.42 | 1.07 |
| **rs17255510** | 22662856 | 0.220 | 0.234 | 0.42 | 0.92 (0.75-1.13) | 0.56 | 0.88 (0.56-1.37) | 0.40 | 0.92 |
| rs2001022 | 22757266 | 0.048 | 0.040 | 0.34 | 1.21 (0.81-1.83) | 0.99 | 1.01 (0.44-2.33) | 0.20 | 0.77 |
| rs11626312 | 22851152 | 0.075 | 0.059 | 0.13 | 1.29 (0.93-1.81) | 0.18 | 0.58 (0.26-1.29) | 0.78 | 0.96 |
| **rs11157573** | 22889777 | 0.180 | 0.178 | 0.93 | 1.01 (0.81-1.26) | 0.09 | 1.46 (0.94-2.26) | 0.48 | 0.93 |
| rs8021297 | 22905065 | 0.473 | 0.490 | 0.43 | 0.93 (0.79-1.11) | 0.14 | 0.76 (0.53-1.09) | 0.53 | 0.95 |
| rs2254272 | 22942890 | 0.322 | 0.303 | 0.35 | 1.09 (0.91-1.31) | 0.17 | 1.30 (0.90-1.89) | 0.77 | 1.03 |
| rs1154155 | 23002684 | 0.196 | 0.190 | 0.72 | 1.04 (0.84-1.29) | 0.56 | 0.87 (0.55-1.38) | 0.76 | 0.97 |
| rs1263655 | 23018822 | 0.241 | 0.261 | 0.27 | 0.90 (0.74-1.09) | 0.97 | 0.99 (0.65-1.51) | 0.76 | 1.03 |
| rs1263656 | 23019164 | 0.336 | 0.340 | 0.85 | 0.98 (0.82-1.17) | 0.71 | 1.07 (0.74-1.56) | 0.86 | 0.99 |
| rs8572 | 23236524 | 0.132 | 0.135 | 0.83 | 0.97 (0.76-1.25) | 0.09 | 0.59 (0.32-1.09) | 0.02 | 0.76 |
| rs11622421 | 23332272 | 0.357 | 0.361 | 0.86 | 0.99 (0.83-1.17) | 0.98 | 1.00 (0.69-1.45) | 0.90 | 1.01 |
| rs12879543 | 23340024 | 0.359 | 0.367 | 0.73 | 0.97 (0.81-1.15) | 0.93 | 0.98 (0.68-1.43) | 0.95 | 1.01 |
| rs8005677 | 23376678 | 0.424 | 0.410 | 0.50 | 1.06 (0.90-1.26) | 0.57 | 1.11 (0.77-1.59) | 0.98 | 1.00 |
